# Evaluation of Socio‐Technical Mechanisms Shaping AI Scribe Documentation Failures: A Netnographic Study

**DOI:** 10.1111/jep.70554

**Published:** 2026-08-13

**Authors:** Samuel Atiku, Kehinde Owolanke, Olufisayo Olakotan

**Affiliations:** ^1^ Digital Technology and Innovation University of Staffordshire Stafford UK; ^2^ Research Services Aston University Birmingham Birmingham UK; ^3^ Department of Obstetrics and Gynaecology Mersey and West Lancashire NHS Trust Liverpool UK; ^4^ Department of Neonatology, Women and Children's Directorate University Hospitals Leicester NHS Trust Leicester UK

**Keywords:** artificial intelligence scribes, clinical documentation, netnography, socio‐technical systems, workflow integration

## Abstract

**Background:**

Artificial Intelligence (AI) scribes are increasingly adopted to address electronic health record (EHR) documentation burden. Although early evaluations report perceived efficiency gains and reduced after‐hours work, findings on documentation quality and safety remain mixed. Reported issues, including omissions, attribution mistakes, and hallucinated content, raise concerns about potential clinical, administrative and medico‐legal risks. Existing evaluations largely focus on performance metrics, offering limited insight into the socio‐technical conditions shaping real‐world experiences and outcomes.

**Aim:**

To examine how interacting socio‐technical conditions influence AI scribe use, reported problems and associated risk implications in clinical documentation.

**Methodology:**

A netnographic analysis was conducted of 2267 relevant data segments from 952 documents across 162 Reddit threads (2023–2025) drawn from clinician‐oriented communities. Data were collected using a structured query design via the Python Reddit API Wrapper. Data were analysed using an inductive–abductive qualitative approach and organised through a socio‐technical lens across technology, organisation, person and environment domains.

**Results:**

Contributors' accounts suggested that reported AI scribe problems were associated with interacting technological constraints, including template rigidity, integration gaps and reliability issues; organisational governance and billing pressures; environmental time constraints; and individual verification practices. These conditions appeared to operate through three mediating mechanisms: adoption and configuration practices, workflow coupling and documentation targets. Reported problems included content‐related issues, such as misattribution, hallucinations and omissions, as well as workflow disruptions, including latency, crashes and copy‐and‐paste friction. Clinicians described potential clinical, administrative and medico‐legal risks as contingent on integration quality, governance clarity and review capacity.

**Conclusion:**

AI scribe safety is not solely a function of model accuracy. The findings suggest that documentation problems may arise through socio‐technical interactions that influence whether errors are identified, corrected or carried forward. Safe deployment requires strengthening integration, governance and verification processes alongside technical performance.

## 1 Introduction

Electronic health record (EHR) documentation burden extends beyond time spent typing, encompassing cognitive load, administrative complexity and workflow fragmentation that frequently spill beyond scheduled encounters and reshape care delivery [1, 2]. Documentation burden has been inconsistently conceptualised and measured, often operationalised narrowly as time or effort, with fewer than half of studies assessing associations with burnout [1, 2, 3]. Objective log‐file analyses nevertheless illustrate the scale of this work, with ambulatory physicians spending substantial time in the EHR even on days without scheduled appointments and continuing documentation after hours on clinic days [4]. Burden is further amplified by fragmented information infrastructures—where documentation is distributed across paper, EHR, and ancillary systems and by widespread reuse of copied text, which can introduce inefficiencies and safety concerns [5].

Against this backdrop, Artificial Intelligence (AI) scribes combining automated speech recognition (ASR), natural language processing (NLP) and large language models (LLMs) to generate clinical notes from clinician–patient conversations—are being adopted as a pragmatic response to workload and burnout pressures [6, 7, 8]. Early evaluations report perceived workflow benefits, including improved perceived ease of documentation, higher likelihood of completing notes during or shortly after encounters, and reductions in documentation burden and after‐hours work [6, 7, 9, 10]. Qualitative accounts also suggest potential relational benefits, with some clinicians reporting greater presence and satisfaction during visits [11]. However, reported gains are not uniform, and benefits can depend on implementation design, integration into local workflows, and the extent of required post‐generation review [6, 12, 13].

At the same time, evaluations of AI scribes show mixed results on usability and documentation quality. Studies using structured rubrics and note‐quality instruments report that tools can generate notes quickly yet still produce recurrent errors, particularly in complex encounters (e.g., multi‐speaker interactions), including omissions, structural problems, and attribution mistakes [6, 12, 14, 15]. Across platforms, transcript‐level inaccuracies may propagate into downstream clinical notes, and LLM‐enabled generation can introduce additional risks such as hallucinated or contextually misinterpreted content [6, 15, 16]. These limitations matter because documentation failures can create safety, administrative and medico‐legal risk specially when scaled across high‐volume clinical settings and may be exacerbated by time pressure and automation bias during routine verification [16, 17].

Importantly, these risks rarely arise from model behaviour alone. They emerge from interactions among system capabilities, configuration choices, organisational governance, clinician practices and environmental constraints [18, 19]. Socio‐technical frameworks for health information technology (HIT) emphasise that such interactions can produce unintended consequences and complicate attribution of failure and harm [20, 21]. Yet most AI scribe evaluations focus on performance and time outcomes, offering limited insight into the real‐world socio‐technical conditions that shape when and how documentation failures emerge and become consequential in real‐world settings.

Therefore, this study aims to examine how interacting socio‐technical conditions shape the real‐world use, failure modes, and risk implications of AI scribes in clinical documentation. Drawing on a netnographic analysis of clinician discourse, we distinguish between (1) work‐system conditions across technology, organisation, person, and environment domains; (2) mediating mechanisms through which these conditions structure everyday practice; and (3) observable failure modes and harm pathways. By specifying how configuration practices, workflow coupling and documentation targets influence whether inaccuracies are corrected, tolerated, or amplified, this study reframes AI scribe safety as a function of implementation ecology rather than model accuracy alone.

## 2 Methodology

### 2.1 Netnographic Approach

We adopted netnography to examine how clinicians narrate, negotiate and normalise AI scribe use within online professional communities, treating Reddit threads as situated cultural texts rather than population‐level evidence [22, 23, 24]. This approach recognises that platform discourse is shaped by community norms, moderation, and algorithmic visibility and must therefore be interpreted in context rather than abstracted as neutral ‘big data’ [25, 26]. The study analyses naturally occurring professional commentary, not a systematic or representative sample of verified physicians. It identifies how documentation failures, adaptations, and mitigations were described in spontaneous discourse. Accordingly, reported experiences, perceived risks, and potential harm pathways are distinguished from confirmed clinical harms, which this design cannot verify.

### 2.2 Data Source and Sampling Frame

We performed a retrospective, automated content analysis of publicly available discussions from four clinician‐oriented subreddits (r/medicine, r/Residency, r/FamilyMedicine, r/emergencymedicine). These communities were purposively selected to increase the likelihood that retrieved texts would reference real clinical documentation workflows and implementation contexts relevant to AI scribe tools. This targeted sampling is consistent with guidance that automated text analysis requires problem‐specific corpus design and careful construct alignment.

Reddit provides access to candid, naturally occurring professional discourse across a broader range of implementation contexts than is typically available in single‐site qualitative studies. However, these self‐reported narratives are subject to self‐selection, likely overrepresent technologically engaged users and unusually positive or negative experiences and arise through asynchronous exchanges. Accordingly, the analysis characterises public professional interpretations of AI scribe use rather than claiming that the findings are universally representative or direct evidence of effects in clinical practice.

### 2.3 API Access and Search Strategy

Data were collected using the Python Reddit API Wrapper (PRAW), which provides programmatic access to subreddit search results, submissions, and comment structures. We queried each subreddit using subreddit search (query = q, sort = ‘new’, time_filter = ‘all’, limit = 120) to obtain a recent‐first stream of submissions matching each query string, using PRAW‐supported search parameters to control sort order and time filtering.

The query set was generated in three layers to enhance recall: (i) concept phrases about ambient/AI documentation, (ii) vendor phrases and (iii) vendor–safety combinations of the form “<VENDOR_QUERY> <SAFETY_TAIL>” (e.g., ‘nuance dax’ privacy). This design was used to capture both general discourse and vendor‐specific experiences, while deliberately enriching retrieval for safety‐relevant content (e.g., hallucination, privacy, consent, workflow integration, data loss) aligned with the study's research questions. Quoted phrases were used to express phrase‐level matching under the platform search syntax as implemented through PRAW. Duplicate query strings were removed while preserving order to reduce redundant API calls and mitigate rate‐limit exposure without narrowing conceptual coverage.

The search used 93 deduplicated query strings combining general AI scribe terms, vendor names, and safety and workflow terms. Concept phrases comprised 'AI scribe', ‘ambient scribe’ and ‘ambient clinical documentation’. Ten vendor phrases named DAX Copilot, Dragon Ambient, Abridge, Suki, Nabla, Heidi Health, Freed, Amazon HealthScribe, Augmedix and Notable. Eighty further strings paired each vendor phrase with one of eight safety and workflow terms, error, wrong, hallucination, omitted, privacy, workflow, integration and consent, which deliberately enriched retrieval for safety‐relevant discussion. Vocabulary covering medication and allergy errors, data loss, note bloat, editing and verification entered the pipeline at the screening stage, through the lexicons described below, rather than as search strings.

Searches ran in each subreddit through Reddit search, sorted newest first with the time filter set to all, and retrieved posts and comments were then filtered locally by creation timestamp so that only material within the study window was retained. Retrieval drew up to 120 submissions per query, and comment retrieval was capped at 250 comments per post. The pipeline processed the window through 36 monthly bins, used for checkpointing and monitoring rather than as a sampling frame. Deleted or removed comments and content authored by AutoModerator were excluded at source. Reddit search is probabilistic rather than exhaustive, so the corpus represents a purposive slice of professional discourse rather than a census of every relevant thread. That constraint sits comfortably with the netnographic aim. Depth mattered more than completeness.

### 2.4 Temporal Inclusion and Month Binning

We defined an analytic window spanning 1 January 2023 to 31 December 2025 (UTC) and enforced inclusion by filtering retrieved items using their Unix timestamps (created_utc). This local timestamp filtering was used because PRAW search provides coarse time filters rather than deterministic absolute date constraints across multi‐year windows. The script also constructed explicit month bins and applied month‐level guards to support systematic processing and monitoring over time.

### 2.5 Comment Retrieval and Two‐Pass Design

For each eligible submission, comments were retrieved via the CommentForest and expanded using replace_more (limit = …) to resolve placeholder nodes while bounding the retrieval cost. A two‐pass design was implemented: Pass 1 extracted submission text and (primarily) top‐level comments for broad coverage; Pass 2 re‐processed only ‘flagged’ threads (threads containing ≥ 1 failure‐mode label) with deeper comment expansion to increase capture of downstream mitigations and contextual factors.

### 2.6 Document Eligibility and Relevance Screening

Each retrieved submission and comment was treated as a candidate document, with the submission title and body concatenated into a single text. Screening applied three automated gates before any qualitative reading. A length gate removed documents shorter than 120 characters after whitespace normalisation, since fragments of that size rarely carry an interpretable account of practice. A clinical context gate then required at least one token indicating a care setting, such as a named EHR platform, a clinical role, or encounter vocabulary, which reduced contamination from consumer and legal transcription discourse. A relevance gate retained documents matching an anchor phrase for ambient or AI documentation, a named vendor, or a generic AI term co‐occurring with a documentation action term. Comments marked deleted or removed, and posts authored by AutoModerator, were excluded at source. Screening was conservative by design. Some relevant material may have been lost.

### 2.7 Unit of Analysis, the Incident Segment

The unit of analysis was the incident segment, defined as the smallest contiguous passage within an eligible document that describes a discrete reported experience of AI scribe use, namely a documentation failure, a mitigation or verification practice, or an identifiable stage of the documentation workflow. The term incident denotes a reported experience rather than a verified clinical event, and the analysis preserves that distinction throughout. Segments were derived through a rule‐based procedure applied uniformly to every eligible document. Text was first normalised for whitespace, then split at sentence boundaries, line breaks and typographic separators such as hyphenated or bulleted breaks. Fragments shorter than 80 characters were merged with the fragment that followed until the threshold was reached, which prevented orphaned clauses from entering the corpus as free‐standing units.

A candidate segment was retained only where it matched at least one entry in the failure mode, mitigation, or workflow stage lexicons developed for the study. Lexicon matching operated as a screening device rather than as the analysis itself. Qualitative coding proceeded on the full segment text, and lexicon labels remained open to revision during coding. Socio‐technical condition labels were assigned at the same stage to support later analysis, although a condition match alone did not retain a segment. A ceiling of eight segments per document stopped long or repetitive posts from dominating the corpus. Segmenting below the document level mattered because contributors often narrate several distinct experiences within one post, and document‐level coding would collapse those accounts into a single unit. Each segment retained its document, thread, and community identifiers so that interpretation could return to the surrounding conversational context at any point.

### 2.8 Deduplication and Corpus Construction

Deduplication operated at two levels. Query strings were deduplicated before retrieval while preserving order, which reduced redundant API calls without narrowing conceptual coverage. Retrieved segments were then deduplicated using a case‐insensitive SHA‐1 hash of the segment text, so passages surfaced by overlapping queries, or reproduced across cross‐posted threads, entered the corpus once. Collection wrote outputs incrementally with checkpointing, allowing interrupted runs to resume without repeating completed work. The construction sequence ran from query retrieval, through temporal filtering, document assembly and screening, incident segmentation, and hash‐based deduplication, to the final analytic dataset. That sequence yielded 162 threads, 952 eligible documents, and 2267 unique incident segments across the four communities. Counts and parameter settings at each stage were logged in run metadata to support auditability.

### 2.9 Inclusion and Exclusion Criteria

Table 1 consolidates the criteria applied at each stage of corpus construction. Criteria were implemented programmatically wherever possible, which fixed them in advance of analysis and removed screener discretion at the retrieval stage. Two caveats apply. Reddit search does not return an exhaustive population of matching content, so the inclusion rules define eligibility within what the platform surfaced rather than within all existing discourse. Automated gates also carry a recall cost, and accounts phrased without any lexicon vocabulary will have been missed.

**Table 1:** Inclusion and exclusion criteria applied at each stage of corpus construction.

Stage	Inclusion criteria	Exclusion criteria
Platform and community	Publicly accessible content in four clinician‐oriented subreddits, r/medicine, r/Residency, r/FamilyMedicine and r/emergencymedicine, each of which passed an automated accessibility check before harvesting began	Private, quarantined, restricted or redirecting communities. The candidate community r/FamilyPractice was replaced with r/FamilyMedicine after redirect checks. Communities without a clinician orientation were not sampled
Search retrieval	Submissions returned by any of 93 unique query strings, namely three ambient documentation concept phrases, ten vendor phrases, and eighty vendor and safety combinations formed by pairing each vendor phrase with one of eight safety terms (error, wrong, hallucination, omitted, privacy, workflow, integration, consent). Results were retrieved newest first, to a ceiling of 120 submissions per query per community	Duplicate query strings, removed before retrieval while preserving order; submissions beyond the 120 per‐query retrieval ceiling
Time window	Content created between 1 January 2023 and 31 December 2025 inclusive (UTC), verified against each item's Unix creation timestamp rather than the platform's coarse time filter	Content created outside the analytic window, regardless of when it was retrieved
Document type and comment expansion	Submissions, with title and body concatenated into one text, and comments at any nesting depth, taken in retrieval order to a ceiling of 250 comments per submission	Comments marked deleted or removed; posts and comments authored by AutoModerator; unresolved placeholder nodes for further comments, which were discarded rather than fetched to bound retrieval cost; comments beyond the per‐thread ceiling
Language	English‐language content, sampled through communities that operate in English	No separate language filter was applied beyond community selection
Document length	Documents of 120 characters or more after whitespace normalisation	Documents below 120 characters, which rarely carry an interpretable account of practice
Clinical context	At least one token from a clinical context lexicon covering named EHR platforms (Epic, Cerner, Oracle Health, athenahealth, eClinicalWorks, NextGen), clinical roles (physician, doctor, GP, resident, attending), and encounter vocabulary (patient, clinic, visit, encounter, rounds, consult, triage, SOAP, H&P, ED, ER)	Documents discussing AI transcription outside a care setting, legal and consumer dictation among them
Topical relevance	Any of three routes. First, an anchor phrase (AI scribe, ambient scribe, ambient AI, ambient documentation, ambient clinical documentation). Second, one of eighteen named vendor tokens, covering Nuance DAX and DAX Copilot, Dragon Ambient, Abridge, Suki, DeepScribe, Nabla, Tali, Amazon HealthScribe, Augmedix, Notable, Heidi Health, Freed, and EHR‐native ambient products. Third, a generic AI token (AI, GPT, LLM, ChatGPT, Copilot) co‐occurring with a documentation action token (scribe, note, chart, dictation, transcription, SOAP or progress note)	Documents satisfying none of the three relevance routes
Incident segment construction	Document text normalised for whitespace, then split at sentence boundaries, line breaks, and hyphen or bullet separators; fragments shorter than 80 characters merged with the fragment that followed until the threshold was reached	Free‐standing fragments below the 80 character threshold that could not be merged
Incident segment retention	Segments of 80 characters or more matching at least one entry across three screening lexicons, nine failure mode categories (hallucination or fabrication, speaker misattribution, omission, incorrect clinical fact, medication or allergy error, privacy or confidentiality, workflow integration breakdown, latency, outage or data loss, and note bloat or structure), six mitigation categories (verification, editing and correction, workflow adjustment, template and prompting, privacy safeguards, manual fallback), or six workflow stages spanning pre‐encounter preparation to post‐encounter downstream use; a ceiling of eight segments per document applied	Segments matching no lexicon entry; segments beyond the per‐document ceiling of eight
Deduplication	First occurrence of each unique segment, identified by a case‐insensitive SHA‐1 hash of the segment text	Repeat occurrences of an identical segment, including passages surfaced by overlapping queries or reproduced across cross‐posted threads

### 2.10 Rate‐Limit Resilience, Incremental Output, and Ethics

To improve completion reliability under rate limiting, the pipeline inserted fixed sleeps and implemented backoff when encountering TooManyRequests/HTTP 429 responses, consistent with documented behaviour of PRAW under high request volumes. Outputs were written incrementally to CSV to reduce data loss risk during long‐running collection. Usernames and URLs were omitted (de‐identification) to reduce re‐identification risk and align with ethical guidance for internet‐mediated research emphasising harm minimisation and contextual sensitivity.

### 2.11 Ethics and Trustworthiness

We adopted a precautionary ethical stance [27], recognising that public availability does not eliminate the duty to protect contextual integrity [28]. We de‐identified the dataset by removing usernames and direct URLs. To mitigate 'trace‐back' risks, where search engines link quotes back to individuals, we avoided reproducing verbatim long quotations, using paraphrases where necessary [29]. Trustworthiness was supported through a documented audit trail of search terms, inclusion thresholds, and triangulation across submissions and comments to check for thematic consistency [30]. No attempt was made to identify or contact any user, and only openly accessible communities were sampled. Where quotations are reported, they were lightly paraphrased or blended to preserve meaning while reducing searchability, and temporal, institutional, and vendor‐identifying details were removed where not analytically load‐bearing. The same treatment was applied to the illustrative extracts presented in the appendices.

### 2.12 Data Analysis

Data were analysed using an inductive–abductive qualitative approach. Analysis began with open coding to identify recurrent descriptions of AI scribe use, adaptations to clinical work, breakdowns and perceived risks in routine practice. Three researchers (OO, AS and KO) contributed to the analysis. OO led the initial coding of the full dataset, while AS and KO independently re‐coded full data set to support analytic rigour. OO, KO and AS reviewed the developing coding framework and contributed to the interpretation and refinement of themes.

OO and AS met regularly to compare interpretations, discuss emerging codes, and resolve coding differences through discussion and consensus. Where necessary, unresolved interpretations were discussed with KO. Coding was iterative and comparative, with data segments examined both within and across participant accounts to refine code definitions, identify similarities and differences, and examine deviant or contradictory cases. Analytic memos were maintained throughout the process to document coding decisions, emerging interpretations, questions, and relationships between codes. These memos supported the progressive development of descriptive codes into higher‐order categories and themes. Theme development involved reviewing coded extracts, identifying coherent patterns across the dataset, and refining theme boundaries to ensure that each theme was analytically distinct and grounded in participants' accounts. Candidate themes were reviewed against both the coded extracts and the wider dataset and were revised through discussion among the research team.

As the analysis progressed, recurring interactions were identified between technological features, organisational arrangements, clinician practices, and the physical and temporal conditions of clinical work. These patterns supported the abductive adoption of a socio‐technical lens. This framing was not specified a priori but developed through sustained engagement with the data to strengthen analytic coherence. Following theme development, the themes were organised across four work‐system domains technology, organisation, person, and environment according to the primary locus of influence within each account, such as system behaviour, institutional arrangements, individual practice, or physical and temporal working conditions. Where findings crossed domains, these boundary‐spanning relationships were retained. The domains were therefore used as an organising framework rather than as a formal coding structure or a newly proposed conceptual model.

### 2.13 Reflexivity

Reflexivity was incorporated throughout the analytic process. The researchers' disciplinary backgrounds and prior knowledge of digital health and AI‐enabled documentation informed the questions brought to the data, particularly the attention given to how AI scribes were used, adapted, and understood in routine clinical practice. The research team recognised that this expertise could also shape interpretation. Potential assumptions and interpretive biases were examined through analytic memo‐writing, independent coding, regular comparison of interpretations, discussion of alternative explanations, and attention to deviant or contradictory accounts. Candidate themes were reviewed and refined collaboratively to ensure that they remained clearly defined, internally consistent, and grounded in participants' accounts rather than in the researchers' prior expectations.

## 3 Results

**Figure 1:**
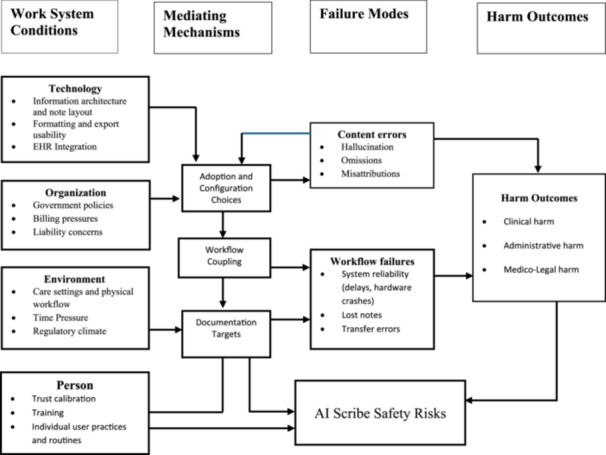
Socio‐technical framework of AI scribe safety failures.

The final dataset comprised 2267 incident segments from 952 documents across 162 threads. Incidents were concentrated in 2025 (1,484; 65.5%), followed by 2024 (682; 30.1%) and 2023 (101; 4.5%), indicating a marked recency skew. By subreddit, incidents were most frequent in r/FamilyMedicine (1,045; 46.1%), followed by r/medicine (632; 27.9%), r/emergencymedicine (364; 16.1%), and r/Residency (226; 10.0%). Our findings distinguish between work‐system conditions (Technology, Organisation, Environment, Person), mediating mechanisms through which those conditions shape practice, and failure modes and harms (outcomes) Figure 1. Conditions provide structural inputs; mechanisms determine whether inaccuracies are intercepted or propagated; outcomes reflect observable breakdowns and consequences. This distinction explains why similar AI scribe tools can yield different safety outcomes across settings: identical technical limitations may be stabilised or amplified depending on configuration stability, workflow coupling, documentation targets and verification capacity. Appendices A, B, C, D, E, F, G, H present additional meaning‐preserving paraphrased evidence supporting the socio‐technical domains and mediating mechanisms identified in the analysis. Distinctive wording and contextual details were generalised to reduce the risk of search‐based traceability.

### 3.1 Technology

Multiple technology‐level usability issues associated with AI scribe use were reported. These centred on the structure of AI‐generated notes and the constraints imposed by rigid documentation formats within the EHR. Participants described tensions between the generated note structure and their preferred documentation styles. One clinician pointed to template rigidity, particularly restriction to the Subjective, Objective, Assessment, Plan (SOAP) format, stating, *‘Freed AI is very simple, but being limited to SOAP feels restrictive’ (P1)*. Another noted, *‘As a primary care physician (PCP), I don't want to be confined to specific note formats’ (P2)*. The need to adapt generated notes prior to EHR entry was also evident, with one participant explaining, *‘I would never copy the full note into the EHR without editing it first; it needs to match my group's format’ (P3)*.

Participants described copy‐and‐transfer processes as a source of friction. One clinician explained that ‘*The SOAP note sections are separated… which makes it frustrating… to copy all the parts I need’ (P4)*. Formatting instability during transfer was repeatedly noted, particularly in Epic, with one participant stating that *‘The formatting does not transfer properly into Epic, and bolded text appears with unusual markup around each bolded word’ (P5)*. Another described different outcomes depending on how copying was performed, observing that *‘When I use the “copy” button in Heidi, the formatting becomes distorted, but if I select everything manually and then copy it, the formatting transfers correctly’ (P6)*. Similar manual workarounds were described in other systems, including Cerner, where one clinician noted, *‘I use copy and paste. Since I'm on Cerner, I have to rely on the keyboard shortcut’ (P7)*.

Integration with the EHR was described as an important usability consideration. Several clinicians highlighted limitations in how AI scribes interacted with structured components of the EHR, with one noting that *‘They handle narrative documentation well, but they do not usually populate the discrete data fields required by EHRs’ (P8)*. Lack of direct integration was frequently cited as a drawback, even when overall note quality was viewed positively. One participant remarked that ‘*Doximity's scribe is fairly good, but its biggest drawback is the lack of electronic medical record (EMR) integration’ (P9)*. While another similarly noted, *‘I liked Freed AI, but it is not yet integrated with any EHRs’ (P10)*.

### 3.2 Organisation

Organisational conditions surrounding AI scribe use centred on formal governance processes shaping adoption and evaluation during trial periods. Clinicians described engagement with AI scribes within institutional decision‐making structures rather than as independently adopted tools. Adoption commonly occurred through structured hospital trials, with participants reporting experience across multiple solutions. One participant stated, *‘The three solutions we tested were Twofold Health, Freed, and Heidi’ (C1)*. Another noted, ‘*My hospital has been piloting Heidi, and I have used it extensively in the emergency department (ED)’ (C2)*. However, some clinicians reported that trial periods provided limited time for evaluation, stating that they *‘Did not have sufficient time to fully assess the tools before decisions were made’ (C3)*. Evaluation was described as extending beyond purely technical performance. One participant noted time constraints during trials, stating, *‘That's true; there was not enough time during the trial period’ (C4)*. Another emphasised that assessment involved multiple considerations, stating, *‘Most importantly, the discussions focused on pricing, quality, usability, and compliance’ (C5)*.

Billing‐related documentation structures and productivity incentives were described as shaping how AI‐generated notes were organised and reviewed. Clinicians highlighted features that structured documentation around medical decision‐making (MDM) and diagnosis‐linked coding, including ED MDM–style Assessment and Plan (A&P) templates and diagnosis‐aware notes that populate problem lists within billing‐relevant sections. One participant stated, *‘It includes an option to generate an ED MDM 2022‐style A&P’ (C6)*. Another noted, ‘*Diagnosis‐aware notes (DAN), Epic's latest diagnosis SmartLink, convert documentation into an automatically populated problem list within the MDM section’ (C7)*. Productivity models were also described as influencing review practices, with proofreading and editing characterised as disincentivised in systems that reward productivity above everything else. One participant stated, *‘Proofreading and editing are discouraged in a model that prioritises productivity over everything else’ (C8)*.

Concerns were raised about institutional control over patient data, legal responsibility, and privacy risk. Clinicians expressed uncertainty about institutional rights over patient data and whether appropriate agreements were in place, with one stating, *‘As a postgraduate year 4 in psychiatry, the hospital owns the patient data, but there is no agreement in place, creating a potential Health Insurance Portability and Accountability (HIPAA) concern’ (C9)*. Participants emphasised that legal and privacy obligations remained unchanged with AI use, even for those without technical expertise, noting, *‘I am not particularly tech‐savvy; however, all healthcare and privacy laws still remain applicable’ (C2)*. Clinicians also described limiting what they entered into AI systems, including avoiding identifiable information, with one stating, *‘I do not provide consent, and I also avoid entering any information that could reasonably connect the dictation to a specific patient’ (C4)*.

### 3.3 Environment

Participants described environmental conditions, particularly the clinical setting and physical workflow, as influencing how AI scribes were used in practice. These factors shaped where AI scribes were perceived as feasible and how they were incorporated into routine clinical work. Clinicians described use in outpatient clinics and ED, with one noting, *‘We currently use DAX Copilot only in outpatient settings and the ED’ (E1)*. Another added that they had *‘Used it extensively within the ED’ (E2)*. One participant emphasised the value of systems described as *‘Genuinely incorporating ED‐specific workflows and being fully customised’ (E3)*. In contrast, clinicians working in more mobile settings raised questions about how AI scribes functioned when moving between rooms, with one noting that *‘We move between rooms and complete the notes afterwards’ (E4)*, highlighting differences in environmental fit.

Time pressure emerged as an environmental constraint shaping how AI scribes were used in practice. Clinicians described documentation workload as embedded in high‐throughput clinical environments, with one noting that *‘Documentation had been taking up too much of my time’ (E5)*. AI scribes were used within this time‐pressured context, but time savings were often redistributed to reviewing AI output, as one clinician acknowledged that ‘*It still makes errors, so I have to go back and make edits’ (E6)*. In high‐volume settings, however, the pace of work limited the feasibility of a thorough review. One participant observed that although *‘Each note needs to be reviewed carefully, in practice this does not always occur’ (E7)*, indicating that environmental time pressures could constrain verification and allow errors to persist.

### 3.4 Person

At the person level, clinicians' use of AI scribes was shaped by trust calibration, skill development, and individual user practices. Clinicians described calibrating their reliance on AI scribes through personal responsibility, experiential learning, and informal peer support. Professional accountability was repeatedly emphasised, with one participant questioning how responsibility should be understood when errors occur, asking, *‘Is the responsibility yours for not reviewing the note carefully enough, or does it lie with the AI developer for the natural language processing error?’ (D1)*. Several clinicians highlighted the importance of learning how to prompt, configure, and interact with AI systems effectively. One participant explained that *‘It depends on how you script it and how well you understand the AI’ (D2)*, indicating that competence developed through hands‐on use. Informal learning within teams also supported this process, with one participant noting that *‘My group has had considerable success using AI because informatics experts within the team taught us how to optimise it’ (D3)*.

Day‐to‐day use of AI scribes involved active review and correction of AI‐generated documentation. Clinicians described routinely reading through notes and correcting errors or omissions, with one stating, *‘I still review every note and correct mistakes, missing details, or dates that were not captured for some reason’ (D4)*. Some participants described interacting directly with the system to make amendments, with one noting that *‘You can speak to the AI and ask it to revise the chart if you notice anything you are not satisfied with’ (D5)*. Consent handling was also described as part of individual workflow routines during patient encounters. Clinicians reported incorporating verbal explanations into their practice, with one stating, *‘We were given a verbal consent script to use; I explain it, the patient agrees, and then I begin the scribe recording’ (D6)*. Others noted that consent discussions could take *“‘At least thirty seconds, and sometimes involve much longer conversations’ (D7)*, indicating that obtaining consent formed part of clinicians' routine practice when using AI scribes.

### 3.5 Mediating Mechanisms

Three recurring mechanisms, adoption and configuration practices, workflow coupling, and documentation targets, structured how technology, organisation, person and environment (TOP‐E) conditions translated into documentation outcomes. These mechanisms determine whether system limitations (e.g., template rigidity, weak integration, latency) are corrected or amplified into record‐level errors and workflow failures, with downstream clinical, administrative and medico‐legal consequences.

### 3.6 Adoption and Configuration Practices

Adoption and use of AI scribes involved ongoing configuration work and adjustments to how systems handled content and formatting. Participants described actively modifying AI‐generated output to manage excessive detail, particularly in the history of present illness (HPI). One clinician stated, *‘It sometimes includes more detail than I need in the HPI, so I shorten it’ (B1)*. Another similarly noted, *‘The main downsides are having to copy and paste into the EHR rather than having direct integration, and that it sometimes includes too much detail in the HPI, so I reduce it’ (B2)*. Effective use was described as linked to optimisation skills and user expertise. Participants emphasised the importance of learning how to configure and script the system effectively, with one stating, *‘I think it largely depends on how you script it and how well you understand the AI’ (B3)*. At the same time, adoption was described as iterative and affected by vendor‐driven formatting changes. While some participants reported incremental improvements, with one stating, *‘They have been adjusting the HPI formatting, and it generally formats reasonably well’ (B4)*. Others described abrupt losses of usability following updates, noting, *‘Unfortunately, recent formatting changes on their side have made it unusable for me’ (B5)*. Participants also described difficulty achieving preferred output formats. One clinician stated, ‘*I also use DAX, but my main concern is that I cannot find the right balance’ (B6)*. While another added, *‘…if I switch it to the ‘concise' format, it produces fragmented sentences and keeps changing the formatting’ (B7)*.

### 3.7 Workflow Coupling

The degree to which AI scribes were embedded into everyday documentation workflows shaped how they were used in practice. Clinicians described simple, tightly coupled workflows in which recording and note generation were straightforward, with one stating, *'I simply switch on the AI scribe and place the phone on the desk beside the computer’ (A1)*. Another described selective use, integrating AI scribes into only part of the workflow, noting, *‘The benefit of having so many options is that I mainly use it for the HPI, while using Epic to dictate or click through the remaining sections’ (A2)*. Some clinicians described using AI scribes to reduce after‐hours documentation burden when tools worked alongside other digital systems. One participant stated, *‘Generating notes in real time helps reduce “pajama time” and works well alongside our other tools, such as Dialer and Doximity Generative Pre‐trained Transformer (GPT)’ (A3)*. Others described delayed sequencing, where AI scribes were used after the encounter to structure dictated notes, with one explaining, *‘I return to my room and dictate a rough SOAP note, which the AI scribe then organises’ (A4)*. Participants also framed direct integration as consequential for adoption, with one explicitly stating, *‘Integrating the tool into the workflow makes a significant difference to adoption’ (A5)*. Another stated that, *‘Direct EHR integration is what turns scribes from helpful tools into genuine time‐saving solutions’ (A6)*.

### 3.8 Documentation Targets

Documentation expectations shaped how clinicians edited and prioritised AI‐generated output. Participants described organisational demands and time constraints influencing what needed to be documented, with one stating, *‘What must be documented, what causes delays, what gets left until the end of the day, what the hospital or group requires, and how much time it ultimately takes’ (F1)*. Clinicians described AI scribes as helping capture details that might otherwise be missed, while also reducing documentation time. One participant noted, ‘There are also many details I might have forgotten to include if the AI had not captured them’ (F2). Another stated, *‘It now completes the HPI, Physical Examination (PE), and A&P and has reduced my clinic documentation time by around 80%’ (F3)*. At the same time, clinicians described actively adjusting output to achieve their preferred balance between completeness and efficiency. One explained, *‘It sometimes includes more detail than I need in the HPI, so I shorten it… I would rather remove extra information than overlook something important’ (F4)*. Others described valuing detailed output for patient‐facing notes, with one stating, *‘DAX is excellent… it captures the details of the history accurately… which patients appreciate when they read their notes and see the level of detail included’ (F5)*.

### 3.9 Reported Limitations and Failure Modes

Reported failure modes refer to breakdowns or limitations in AI Scribe documentation and workflow described in Reddit discussions. These accounts were not independently verified as clinical incidents and should therefore be interpreted as reported experiences and perceived socio‐technical vulnerabilities rather than confirmed failures or harms. Self‐identified clinicians described accuracy problems involving mishearing, incorrect attribution, and hallucinated content that required manual correction. One contributor reported, *‘My DAX would often confuse male and female patients, and I had to review the entire note to correct the use of he and she’ (M1)*. Another described efforts to reduce hallucinations that were not always fully successful, noting, *‘Heidi's templates use prompts designed to prevent this type of hallucination, but it can still occur occasionally’ (M2)*.

These reported issues raised concerns about possible downstream consequences if errors were not identified during clinician review. One contributor stated, *‘I am concerned about how continuity of care may be affected when the AI mishears something and continues based on that error’ (M3)*. However, reported experiences varied across systems and contexts. One contributor reported, *‘I have never experienced or heard of a hallucination occurring in our system’ (M4)*, highlighting variability in reported experiences rather than a uniform pattern of failure.

Contributors also reported that AI‐generated summaries could omit smaller but potentially meaningful aspects of an encounter. One stated, *‘Sometimes a very talkative patient can cause a small detail to be overlooked’ (M5)*. Others described a perceived trade‐off between efficiency and note quality, with one noting, *‘AI‐generated notes save me time when I accept them as they are… however, they are still not as good as the notes I dictate myself’ (M6)*. Reported workflow limitations included slow generation and latency, crashes and lost recordings, manual transfer requirements, and integration problems. Contributors described lost recordings, freezes and crashes that disrupted their documentation workflows. One stated, *‘I have had several visits fail to record because the app crashed during the appointment’ (M7)*. Another described system strain, noting, *‘With that and all the templates, it feels like the computer becomes completely frozen’ (M8)*.

Some contributors described situations in which AI scribes helped maintain documentation flow during system outages, with one noting, *“Maintaining the usual workflow even when the EMR is unavailable” (M8)*. Others described delays in note generation that they considered tolerable when brief, with one clinician stating, “*I wait around 10–15 s for the note to be generated” (M9)*. However, some contributors reported that performance limitations interrupted clinical workflow or displaced documentation work to later in the day. One stated that *“Completing notes could still take more than 20 min at the end of the day” (M10)*. Another described the system as *“Resource‐intensive, sometimes slightly slow on a tablet or phone, and much too demanding for a typical slow clinic computer” (M11)*. These accounts illustrate differing user experiences and perceived workflow vulnerabilities. They do not establish the prevalence, frequency, or severity of such problems across AI scribe systems or clinical settings.

### 3.10 Perceived Risks and Potential Harm Pathways

The discussions also identified perceived risks and potential pathways through which documentation errors or workflow breakdowns could contribute to clinical, administrative, or medico‐legal consequences if they were not detected and corrected. The data did not provide evidence of independently verified patient harm, confirmed adverse clinical incidents, or audited administrative and legal outcomes. Medico‐legal and governance concerns related primarily to privacy, consent, regulatory compliance, institutional authorisation, and professional accountability. Contributors questioned the clarity and interpretation of vendor compliance claims. One stated, *‘We cannot guarantee that we are “HIPPA” compliant, but we do comply with HIPAA, General Data Protection Regulation (GDPR), Personal Information Protection and Electronic Documents Act (PIPEDA), and Australian Privacy Principles (APP) requirements’ (H1)*. Another asked, *‘Which framework do you use to assess their privacy policies and compliance?’ (H2)*. Organisational restrictions were also discussed, with one contributor noting,*‘Some programmes still resist AI scribes because of privacy requirements’ (H3)*. Institutional authorisation was emphasised by another contributor, who stated, *‘Unless your employer has already reviewed the tool and established the appropriate Business Associate Agreements (BAAs), it is not compliant’ (H4)*. Legal responsibility was described as remaining with clinicians, with one stating, *‘All healthcare and privacy laws continue to apply, just as they always have, whether or not this programme is used’ (H5)*. Consent obligations were also questioned directly, with one contributor asking, *‘Is consent required when using AI?’ (H8)*. Another advised, ‘*Anyone using the tool independently should carefully review its privacy policy’ (H9)*. These discussions reflect contributors' uncertainty and perceived governance vulnerabilities rather than documented legal or regulatory violations.

Potential administrative consequences were discussed in relation to billing and documentation language. One contributor warned, *‘Avoid documenting that a patient is “medically ready for discharge,” as this can lead to an immediate insurance denial’ (H10)*. Others described documentation practices as being shaped by billing‐oriented system design, observing that ‘*The balance between documentation speed and detail reflects a broader problem with current EHR systems, as they are designed more around billing compliance than clinical efficiency or educational value’ (H11)*. These accounts indicate perceived administrative risk pathways but do not demonstrate confirmed insurance denials or other administrative consequences attributable to AI scribe use.

Potential clinical risks were discussed in relation to record accuracy and the incorporation of clinical reasoning functions into AI scribe systems. One contributor reported that their system could generate reasoning elements, stating, *‘We also produce differential diagnoses, testing suggestions, and treatment recommendations based on the transcript…’ (H12)*. Another emphasised the need for additional clinician oversight, stating, ‘*When clinical decision‐making is built into AI scribes, the output will need to be checked very carefully’ (H13)*. These comments express perceived patient‐safety concerns and highlight possible pathways through which uncorrected content could influence documentation or decision‐making. However, they do not constitute evidence that these pathways resulted in confirmed patient harm.

## 4 Discussion

These findings advance understanding of AI scribe safety by demonstrating that documentation failures are not merely properties of model accuracy, but are shaped by three mediating mechanisms through which socio‐technical conditions translate into harm: (1) adoption and configuration practices, (2) workflow coupling and (3) documentation targets. Rather than treating errors as isolated technical defects, our analysis shows how they emerge from dynamic interactions between system capabilities, organisational incentives, clinician practices, and environmental constraints.

These mechanisms were shaped by conditions across four work‐system domains technology, organisation, person and environment. Technology conditions influenced configuration stability and integration quality; organisational incentives defined documentation targets and governance boundaries; individual calibration shaped verification behaviour and reliance; and environmental pressures constrained review capacity in time‐sensitive clinical settings. This integration clarifies how domain‐level conditions are not independent contributors to risk, but operate through identifiable pathways that structure when and how documentation failures become consequential.

First, adoption and configuration practices influenced the structure, tone, and detail of AI‐generated notes. Clinicians actively tuned outputs, adjusting verbosity, prompting behaviour, and formatting, to align with local expectations. However, unstable vendor updates, limited template flexibility, or poorly calibrated settings increased vulnerability to omissions, hallucinations, and structural misalignment. This reframes ‘accuracy’ as partially contingent on configuration ecology rather than solely on underlying model performance. Prior evaluations have documented usability variation and recurrent note‐quality issues [11, 31], but our findings extend this by showing how configuration instability becomes a pathway through which content errors are introduced or normalised in routine practice.

Second, workflow coupling determined whether errors were intercepted or propagated. Where AI scribes were tightly integrated into EHR systems, documentation review could occur within a stable workflow. In contrast, weak integration particularly copy–paste transfers across systems, introduced friction, formatting degradation, delays and opportunities for error propagation. Existing studies note integration challenges and post‐generation editing burdens [9, 32, 33], yet our findings clarify the mechanism: coupling strength shapes error detection probability. In loosely coupled workflows, verification becomes discretionary and time‐pressured, increasing the likelihood that inaccuracies persist into the legal medical record.

Third, documentation targets defined by billing rules, medico‐legal expectations, and professional norms—structured how clinicians evaluated and edited AI output. Clinicians did not simply accept or reject notes; they trimmed, expanded or reshaped them to satisfy coding, compliance or learning expectations. This finding complicates dominant narratives that frame AI scribes purely as efficiency tools. Policy analyses show that documentation increasingly serves reimbursement and compliance imperatives [34, 35], and our data suggest that these institutional targets actively reshape how AI‐generated text is scrutinised and modified. Efficiency gains reported in deployment studies [36, 37] may therefore be redistributed into review and reconciliation work when documentation standards remain high.

Situating these mechanisms within the broader literature, prior work has demonstrated that AI scribes can reduce documentation time while generating new forms of review burden and integration friction [9, 32, 36]. Evaluations also document persistent transcript and note‐level inaccuracies, including omissions and hallucinations [6, 15, 38]. Rather than reiterating error rates, our contribution lies in explaining how such errors become consequential: socio‐technical conditions influence whether inaccuracies are corrected, tolerated, or amplified. This perspective aligns with broader HIT safety scholarship emphasising that unintended consequences arise from interactions across technical and organisational domains [16], but applies that logic specifically to ambient documentation systems.

Our findings also illuminate the three harm pathways described by clinicians—clinical, administrative and medico‐legal. Clinical risks emerged when AI output extended beyond transcription into synthesis or reasoning, particularly under time pressure. Administrative harms reflected documentation–billing coupling, where phrasing or structural choices could influence reimbursement. Medico‐legal concerns centred on consent ambiguity, governance gaps and accountability boundaries, echoing calls for clearer policy frameworks around ambient listening and draft‐record management [39, 40]. Importantly, clinicians did not describe harm as inevitable. Rather, risks were viewed as contingent on governance clarity, integration quality, and the reliability of human review processes.

Together, these findings suggest that improving AI scribe safety requires more than improving model accuracy. Interventions may need to target configuration stability, integration architecture, workflow design and governance transparency. In particular, strengthening workflow coupling, clarifying documentation targets, and formalising review expectations may reduce the likelihood that low‐level inaccuracies escalate into downstream harm. This shifts the implementation question from ‘Does the tool work?’ to ‘Under what socio‐technical conditions does it work safely? Future research should refine this framework by modelling feedback loops among configuration practices, workflow coupling, documentation targets, and verification behaviour. Incorporating measurable indicators of integration strength, configuration stability, and governance clarity would improve explanatory precision and help identify modifiable system‐level drivers of clinical, administrative and medico‐legal risk.

## 5 Conclusion

AI scribe safety cannot be assessed through accuracy metrics alone. Based on self‐reported and independently unverifiable Reddit discussions, this study suggests that documentation reliability is shaped not only by tool performance but also by how systems are configured, integrated, governed, and reviewed in practice. The findings identify perceived risks and potential pathways through which errors may persist or become embedded in the medical record when appropriate safeguards are absent; they do not establish that verified clinical incidents or patient harms occurred. As AI scribes are increasingly adopted across healthcare settings, evaluation should therefore consider technical performance alongside integration architecture, clinician verification practices, governance arrangements and organisational accountability. Sustainable deployment is likely to depend on aligning technological capability with socio‐technical safeguards that support the detection, correction, and containment of documentation errors.

## 6 Study Strength and Limitations

A key strength of this study is its theoretically informed netnographic design, capturing real‐world clinician experiences across diverse settings and vendors over 3 years. Incident segmentation, deduplication, and taxonomy‐guided labelling enhanced analytic rigour and transparency. The inductive–abductive socio‐technical approach allowed analytic categories to remain grounded in contributors' accounts while using theory to explain interactions between technological, organisational, individual and environmental conditions. This approach moved beyond isolated performance metrics to identify possible mechanisms linking work‐system conditions, reported failures and perceived risks. The emergent framework also offered a flexible way of organising complex, boundary‐spanning findings without imposing a predetermined model on the data.

However, because the socio‐technical framework emerged through interpretation of the same dataset, its development may have been influenced by researchers' prior knowledge, analytic assumptions, and decisions about how data were assigned to domains. Abductive interpretation can also create a risk of retrospectively fitting observations to a theoretically coherent explanation, particularly where findings overlap across domains. The framework should therefore be regarded as an interpretive and organising framework rather than a validated conceptual model, and its explanatory value requires examination in other datasets and clinical settings.

Other limitations include reliance on self‐selected Reddit contributors, unverifiable clinical roles, and narrated rather than independently verified clinical incidents or audited patient records. Consequently, the findings represent reported experiences, perceived risks, and potential harm pathways rather than confirmed failures, adverse events or patient harms. The study cannot establish the occurrence, prevalence, severity or causal consequences of the reported issues. Algorithmic visibility and search constraints may also have shaped data capture. To reduce search‐based traceability, verbatim excerpts were replaced with meaning‐preserving paraphrases and distinctive contextual details were generalised; however, residual identification risk cannot be entirely eliminated, and paraphrasing may have reduced some linguistic and contextual nuance. Although monthly binning reduced temporal skew, the findings remain interpretive and explanatory rather than representative or generalisable to population‐level prevalence estimates.

## Author Contributions

O.O. and S.A. conceptualized the study. S.A. conducted the search and retrieved data from Reddit using Python. O.O., S.A. and K.O. analyzed and interpreted the data for the qualitative content analysis. O.O. and S.A. drafted the manuscript, while K.O. critically reviewed and revised the manuscript. O.O. and S.A. contributed equally to the work.

## Funding

The authors have nothing to report.

## Ethics Statement

This study received ethics approval from the College of Engineering and Physical Sciences Research Ethics Committee at Aston University (EPS25013). Only publicly accessible Reddit discussions were included. No usernames, profile information, direct links, or other identifying details are reported. To reduce the risk of search‐based traceability, long verbatim excerpts were replaced with meaning‐preserving paraphrases, quotation marks were removed, and distinctive contextual details were generalised where they were not analytically necessary. The paraphrased material was reviewed to preserve the meaning of contributors' accounts while reducing the likelihood that individual posts or users could be identified through online searches.

## Consent

The authors have nothing to report.

## Conflicts of Interest

The authors declare no conflicts of interest.

## Data Availability

The data sets generated and analysed during the current study are not publicly available due to confidentiality but are available from the corresponding author on reasonable request.

## Appendix A Technology‐Level Conditions

**Table:**

Technology condition	Operational definition used in analysis	Paraphrased illustrative evidence
Content integrity limitations	Errors, hallucinations, misattribution, or failure to clinically restructure evolving encounters.	Contributors reported that systems could make incorrect assumptions about speaker identity, names, or gender and could blur the distinction between patients' statements and clinicians' assessments. Some users found that prompts had to be carefully designed to prevent the model from adding unwanted information. Others described weaknesses in organising clinical information and updating medical decision‐making sections when new information emerged during an encounter.
Formatting and structure mismatch	Rigid templates or structural misalignment with local documentation norms requiring adaptation.	Some tools were described as offering only fixed formats, such as SOAP notes, with limited options for creating or modifying templates. Users also reported that generated notes did not always populate preferred sections, including problem lists. Even positively regarded systems often required repeated template adjustment before the output matched local documentation preferences.
Integration constraints	Lack of native EHR integration requiring manual transfer, or integration that depends on local system configuration.	Several contributors had to transfer generated text manually from a separate application into the electronic health record and then complete diagnoses, examination findings, or coding themselves. The ability to copy, paste, or transfer content varied by EHR version and local configuration. Other contributors described more integrated arrangements in which generated content moved directly into existing templates with little additional interaction.
Reliability and latency	System instability, crashes, recording failures, or performance delays shaped by software and infrastructure.	Users described mobile applications crashing during recording and occasional delays in generating notes. Some considered more advanced reasoning functions better able to coordinate information but slower in practice. Recording and performance problems were also attributed to the device or local computing infrastructure rather than solely to the AI system.

## Appendix B Environmental‐Level Conditions

**Table:**

Environmental sub‐theme	Analytic definition	Paraphrased illustrative evidence
Care setting and physical workflow	How the physical clinical setting, such as emergency care, outpatient practice, room‐to‐room work, and mobile workflows, shapes the feasibility and usefulness of AI scribes.	Contributors described using or trialling AI scribes in outpatient and emergency‐care environments, with some systems adapted to the workflow of particular settings. Clinicians who moved between rooms or completed notes later questioned how well these tools supported highly mobile work. Some experimented with carrying a phone or using a tablet to create a fully mobile documentation workflow.
Mobile versus desktop use and form factor	Constraints introduced by screen size, interface density, and input methods when using AI scribes on phones or tablets rather than desktop workstations.	Mobile applications were useful for capturing encounters, but smaller screens often made reviewing and editing notes easier on a desktop or web interface. Users described some interfaces as cluttered or difficult to operate on phones and tablets, particularly where controls required precise interaction without tactile feedback. Web applications could also perform poorly on slower clinic computers, creating a trade‐off between mobility and processing capacity.
Hardware and device constraints	The influence of local hardware, including computer speed, microphones, tablets, and phones, on AI‐scribe performance and usability.	Performance was reported to improve on faster computers, while some systems could be incorporated without purchasing additional recording equipment. Contributors used a range of devices, including phones, tablets, specialist dictation microphones, and inexpensive USB microphones. Device choice affected ease of transferring content, hardware cost, compatibility with the EHR, and the inconvenience of security checks when devices changed.
Audio capture and examination‐room setup	Environmental factors related to audio recording, microphone placement, and room setup that affect capture quality and workflow speed.	Contributors considered whether phones, computers, or wireless microphones provided the most practical way to capture conversations in examination rooms. Some sought alternative microphone arrangements when their existing setup was inconvenient. Audio playback was viewed as useful for checking uncertain content, although it could slow work in time‐pressured settings.
Language and conversational noise	How multilingual encounters and clinically irrelevant conversation shape transcription quality and editing burden.	Some users reported satisfactory performance during encounters conducted partly or wholly in another language and described translation support as time‐saving. At the same time, systems could capture lengthy conversational material that was not clinically relevant, increasing the need for review and editing.
Scheduling and application‐context mismatch	Environmental mismatches between clinic scheduling systems and AI‐scribe applications that create friction during use.	Contributors reported that appointments created through external or online systems did not always appear automatically in the scribe application and sometimes had to be added manually.

## Appendix C Person‐Level Conditions

**Table:**

Person‐level sub‐issue	Analytic definition	Paraphrased illustrative evidence
Trust and training	How clinicians develop confidence, competence, and appropriate reliance on AI scribes through formal or informal learning, and calibrate trust while retaining professional accountability.	Contributors emphasised that clinicians remained responsible for the final note and for identifying errors, although some questioned how responsibility should be shared with developers when system‐generated mistakes occurred. Effective use was associated with understanding how to prompt and configure the system, sometimes with support from informatics specialists or experienced early adopters. AI‐generated structure could reduce the mental effort of composing sentences, but users still warned against excessive reliance because mistakes remained possible.
User practices	Day‐to‐day behaviours clinicians adopt when using AI scribes, including review and correction routines, consent handling, configuration, and privacy checks.	Clinicians described reading every generated note, correcting errors, restoring missing dates or details, and in some systems verbally instructing the tool to revise the draft. Consent was commonly obtained verbally before recording, sometimes through an institutional script; contributors noted that this could take additional time, especially when patients had questions. Some used transcription for other professional encounters only with consent, while organisations discouraged personally purchased tools because of security and privacy concerns. Users also described reviewing vendor privacy policies before adopting a system.
Norms and expectations	Shared beliefs about acceptable documentation, appropriate reliance on AI, responsibility for errors, and effects on learning, professionalism, work, and wellbeing.	Contributors debated whether AI scribes weaken clinical thinking or instead free clinicians to focus more fully on the encounter. Professional expectations varied: some stressed that trainees should first learn traditional documentation, and many regarded review before signing as non‐negotiable, while others reported managerial pressure to accept imperfect notes. Human scribes were perceived as better at contextual interpretation, whereas AI was valued for consistency and the potential to reduce burnout. The discussions also included concern that increasing automation could eventually threaten some roles.

## Appendix D Organisational‐Level Conditions

**Table:**

Theme	Analytic definition	Paraphrased illustrative evidence
Billing‐driven notes	Clinical documentation that is structured, expanded, or shaped primarily to satisfy reimbursement, coding, or regulatory requirements, rather than to support clinical reasoning, care coordination, or patient understanding. In AI‐supported documentation, this occurs when automated coding, billing optimisation, or organisational revenue incentives influence note content, format, or emphasis.	Contributors linked reduced documentation burden with the possibility of seeing more patients and increasing organisational revenue. AI scribes were described as helping produce required note formats, medical decision‐making sections, time‐based documentation, and suggested diagnosis or procedure codes. Some users valued automated support for coding and billing compliance, while others found these functions inaccurate or unhelpful and disabled them. Participants also noted that changing a diagnosis code could alter related procedure codes, pointers, and note content, showing how billing features can shape the wider record.
Privacy	Concerns about who can access data, where data are stored, server location, third‐party sharing, security, and whether vendors can be trusted with patient information.	Contributors wanted clearer information about data access, storage location, underlying software, server location, and onward sharing with third parties. Some reported intermittent security or connectivity problems in healthcare settings and questioned whether these would affect long‐term use. Perceived weaknesses in vendor advertising or transparency reduced trust in companies handling patient information. Users acknowledged that clinicians might not fully understand the technical system, but maintained that existing privacy duties still applied; cloud‐based storage was seen as a risk that also exists in contemporary EHR infrastructure.

## Appendix E Mediating Mechanism ‐ Adoption and Configuration of AI Scribes

**Table:**

Adoption driver or theme	What the data show	Paraphrased illustrative evidence
Overcrowded market and peer‐led adoption	Clinicians operate in a crowded AI‐scribe marketplace and rely heavily on peer advice and trial‐and‐error when selecting tools.	Contributors described testing numerous products and finding that many relied on similar note formats that did not always match local practice. Peer reviews and recommendations were used to compare mobile platforms, transfer methods, pricing, and the value of using a tool for only selected note sections. Users also exchanged templates and sought advice from colleagues to avoid duplicating configuration work. Some recommended presenting the operational and billing benefits to informatics leaders to support formal organisational approval.
Governance and vendor‐transparency constraints	Adoption is influenced by institutional governance, legal compliance requirements, pricing structures, and perceived vendor transparency or credibility.	Contributors treated the absence of appropriate contractual safeguards, despite claims of regulatory compliance, as a reason for caution. Access to structured outputs could depend on paid plans, which influenced adoption decisions. Some preferred inexpensive or locally hosted tools and manual transfer because this appeared to reduce the need for institutional integration or approval. Participants also expected professional guidance and legal requirements to evolve, potentially requiring future changes to current arrangements.
Pragmatic workflow and EHR fit	Clinicians adopt or reject AI scribes according to how well the tools fit their EHR systems, clinic hardware, and specialty workflows.	Poor integration required clinicians to copy generated content into the EHR manually, whereas direct integration was viewed as a substantial time‐saver. Some contributors considered integration into the note essential to practical usefulness. Adoption was also influenced by whether tools could summarise relevant sections, suggest codes, remain lightweight, and transfer content easily into existing systems.
Configuration as a safety mechanism	Clinicians configure templates, prompts, retention settings, and coding functions to manage risks related to privacy, billing emphasis, verbosity, and accuracy.	Users valued settings that limited transcript retention and automatically captured encounter duration, although these features also linked documentation to billing functions. Contributors adjusted or avoided automated coding when suggested diagnoses were irrelevant, incomplete, or inconsistent with the encounter. Templates and prompts were modified to reduce verbose, repetitive, or inaccurate assessment and plan sections. Some clinicians deliberately summarised the patient's account and plan aloud to improve capture, and consistently treated the generated note as an editable first draft requiring professional verification.

## Appendix F Mediating Mechanism ‐ Workflow Coupling

**Table:**

Workflow‐coupling pattern	Analytic definition	Paraphrased illustrative evidence
Loose coupling: AI outside the EHR with manual transfer	The AI scribe operates as an external drafting or summarisation tool. Clinicians manually transfer content and retain full control over what enters the legal medical record, increasing handoff steps and reducing automated provenance.	Clinicians used transcripts or generated drafts differently depending on workload, but the final record consisted of content they had selected, edited, and entered themselves. Some limited use to selected sections because the cost was difficult to justify for partial functionality. External clinical documents could be added as context for summarisation before a visit, but contributors still emphasised that the clinician should review and edit the resulting note.
Hybrid or selective coupling: AI used alongside existing tools	The AI scribe supports selected sections or tasks while clinicians continue to use dictation or manual entry for others, resulting in partial embedding within the documentation workflow.	Clinicians sometimes retained traditional dictation for complex assessment and plan sections while using the AI for history, background capture, or simpler components. Some kept the scribe running as a backup to capture details they might otherwise miss, even when they usually documented independently. Users could dictate a structured plan to replace the system's proposed content or copy the subjective section while writing the assessment and plan themselves.
Tighter coupling: AI embedded in the documentation flow	The AI scribe is closely integrated into the documentation pipeline, automatically formatting or inserting content into the clinical note, reducing transfer friction but increasing reliance on AI‐generated text in the official record.	Contributors described systems that transferred content directly into the note and automatically organised it into standard clinical formats. Some reported that integrated systems produced acceptable drafts consistently enough to reduce editing. Greater customisation and automatic recognition of relevant phrases were associated with outputs that more closely matched clinicians' preferred style.

## Appendix G Reported Content‐Error Modes

**Table:**

Error theme	Analytic definition	Paraphrased illustrative evidence
Hallucination	AI‐generated content that was not present in the source conversation or clinical encounter but was produced as plausible clinical text, sometimes requiring active mitigation.	Some systems or users applied additional checking and regeneration steps in an attempt to identify unsupported content before finalising a note. Contributors warned that models could misinterpret conversations or add information and therefore required careful review. Prompt and template design was used to reduce unsupported content, although users reported that it could still occur. Experiences varied, with some contributors reporting that they had rarely or never observed this problem in their own system.
Misattribution	Errors in which statements, concerns, or intentions are assigned to the wrong speaker, creating confusion about authorship or meaning within the record.	Contributors described clinician questions or reactions being incorporated into the note as though they reflected the patient's own concerns or views. Some observed incorrect transcription or speaker attribution while watching the system operate. To reduce this problem, users sometimes repeated or summarised the patient's account and the agreed plan near the end of the encounter.
Omission	Failure of the generated note to capture clinically relevant details, qualifiers, or reasoning, resulting in oversimplified or incomplete summaries.	AI summaries could miss smaller details within lengthy or complex conversations, which encouraged some clinicians to keep the tool running as a secondary capture mechanism. Contributors were concerned that highly compressed summaries could remove clinically meaningful context. Although accepting the draft without substantial editing saved time, some regarded the resulting documentation as less complete or lower quality than their own dictated notes.
Incorrect or irrelevant facts	Generated information that is factually inaccurate, clinically inappropriate, or unrelated to the encounter and could mislead downstream readers if not corrected.	Users reported generated diagnoses that did not correspond to the encounter, as well as failure to include diagnoses that had actually been discussed. Some perceived software updates as reducing note accuracy or removing previously useful templates. Persistent errors and awkward wording could remain despite repeated prompt or template adjustment. Systems also occasionally inserted unnecessary personal or contextual details into the history while filtering other conversational material.
Latent versus salient errors	Errors that are difficult to detect because a polished and clinically plausible note can conceal inaccuracies or omissions, requiring deliberate verification.	A well‐structured note could appear credible even when users were uncertain that it accurately reflected the encounter. Targeted audio replay was used to verify questionable passages without reviewing the entire transcript. Contributors maintained that clinician editing remained necessary and that the need to read every draft limited potential time savings. Perceived reliability varied, reinforcing the importance of calibrated rather than automatic trust.

## Appendix H Perceived Risks and Potential Harm Pathways

**Table:**

Theme	Analytic definition	Paraphrased illustrative evidence
Potential clinical harm through compromised record integrity	A perceived pathway through which inaccurate, unsupported, omitted, or misattributed documentation could enter the medical record and potentially influence subsequent clinical interpretation or decision‐making.	One account described a clinically important term being misheard and then repeated in later sections of the note, where it affected discussion of possible medication choices. Contributors also reported irrelevant diagnoses being added and encounter diagnoses being omitted. Some expressed concern that allowing a scribe to generate diagnoses or treatment plans could blur the boundary between documentation support and clinical decision‐making. These accounts describe perceived risk pathways and reported errors, not independently verified patient harm.
Potential administrative consequences of billing‐ and compliance‐driven documentation	A perceived pathway through which wording, verbosity, or billing‐oriented documentation could contribute to claim denials, payer scrutiny, audits, or records that prioritise reimbursement requirements over clinical usefulness.	A contributor warned that particular discharge wording could prompt an insurer to deny payment. Others argued that contemporary documentation systems prioritise billing compliance more strongly than clinical efficiency or educational value. Generated notes were also described as excessively detailed while failing to represent important social circumstances affecting care. These accounts indicate possible administrative consequences but do not verify that AI‐scribe use caused a denial, audit, or other adverse outcome.
Perceived medico‐legal risk and liability anxiety	Concerns that stored recordings, persistent inaccuracies, uncertain regulatory expectations, or the presentation of AI‐generated notes could increase legal exposure.	Contributors worried that retaining encounter recordings for extended periods could create discoverable material that might later be interpreted without sufficient context. Some regarded the medico‐legal implications of recording and reviewing consultations as burdensome or insufficiently resolved. Participants also anticipated that the appearance and content of AI‐assisted records could become relevant in future malpractice proceedings. These statements represent perceived liability concerns rather than documented legal findings or confirmed claims.
